# Assessment of treatment response in cardiac sarcoidosis based on myocardial ^18^F-FDG uptake

**DOI:** 10.3389/fimmu.2023.1286684

**Published:** 2023-11-24

**Authors:** Lukas Frischknecht, Jan Schaab, Eloi Schmauch, Ayla Yalamanoglu, Dennis D. Arnold, Judith Schwaiger, Christiane Gruner, Ronny R. Buechel, Daniel P. Franzen, Antonios G.A. Kolios, Jakob Nilsson

**Affiliations:** ^1^ Department of Immunology, University Hospital Zurich (USZ), Zurich, Switzerland; ^2^ Department of Nuclear Medicine, Cardiac Imaging, University Hospital Zurich, Zurich, Switzerland; ^3^ Broad Institute of MIT and Harvard, Cambridge, MA, United States; ^4^ Artturi Ilmari (A.I) Virtanen Institute, University of Eastern Finland, Kuopio, Finland; ^5^ Computer Science and Artificial Intelligence Laboratory, Massachusetts Institute of Technology, Cambridge, MA, United States; ^6^ Department of Cardiology, University Hospital Zurich, Zurich, Switzerland; ^7^ Department of Pulmonology, University Hospital Zurich, Zurich, Switzerland; ^8^ Department of Dermatology, University Hospital Zurich, Zurich, Switzerland

**Keywords:** cardiac sarcoidosis, myocardial 18F-FDG PET, Anti-TNF, adalimumab, azathioprin, SUVmax, cardiac metabolic activity

## Abstract

**Objective:**

Immunosuppressive therapy for cardiac sarcoidosis (CS) still largely consists of corticosteroid monotherapy. However, high relapse rates after tapering and insufficient efficacy are significant problems. The objective of this study was to investigate the efficacy and safety of non-biological and biological disease-modifying anti-rheumatic drugs (nb/bDMARDs) considering control of myocardial inflammation assessed by ^18^F-fluorodeoxyglucose positron emission tomography/computed tomography (^18^F-FDG PET/CT) of the heart.

**Methods:**

We conducted a retrospective analysis of treatment response to nb/bDMARDs of all CS patients seen in the sarcoidosis center of the University Hospital Zurich between January 2016 and December 2020.

**Results:**

We identified 50 patients with CS. Forty-five patients with at least one follow-up PET/CT scan were followed up for a mean of 20.5 ± 12.8 months. Most of the patients were treated with prednisone and concomitant nb/bDMARDs. At the first follow-up PET/CT scan after approximately 6.7 ± 3 months, only adalimumab showed a significant reduction in cardiac metabolic activity. Furthermore, comparing all serial follow-up PET/CT scans (143), tumor necrosis factor inhibitor (TNFi)-based therapies showed statistically significant better suppression of myocardial ^18^F-FDG uptake compared to other treatment regimens. On the last follow-up, most adalimumab-treated patients were inactive (n = 15, 48%) or remitting (n = 11, 35%), and only five patients (16%) were progressive. TNFi was safe even in patients with severely reduced left ventricular ejection fraction (LVEF), and a significant improvement in LVEF under TNFi treatment was observed.

**Conclusion:**

TNFi shows better control of myocardial inflammation compared to nbDMARDs and corticosteroid monotherapies in patients with CS. TNFi was efficient and safe even in patients with severely reduced LVEF.

## Introduction

Sarcoidosis is an inflammatory disease defined by the presence of granulomatous inflammation in affected organs. In individuals with a chronic disease course, the ongoing inflammation may result in tissue destruction with subsequent scarring and fibrosis (1, 2). In addition to pulmonary involvement being the most commonly affected organ, cardiac involvement is associated with significant morbidity and mortality (3). It has been reported to be clinically apparent in 2%–5% of unselected patients with active sarcoidosis (4). However, systematic evaluation of patients with chronic sarcoidosis with magnetic resonance imaging (MRI) as well as data from autopsy studies suggested that cardiac involvement is much more prevalent with a reported incidence of 25%–80% (3, 5, 6). The introduction of ^18^F-fluorodeoxyglucose positron emission tomography/computed tomography (^18^F-FDG PET/CT) for the detection of cardiac sarcoidosis (CS) has further improved diagnostic accuracy and is currently the most sensitive technique to assess CS inflammatory activity (7, 8). Therefore, cardiac ^18^F-FDG PET/CT has been added to the Japan Circulation Society (JCS) criteria for the diagnosis of CS (9, 10). Furthermore, sequential ^18^F-FDG PET/CT scans can provide important information in the evaluation of treatment response to immunosuppressive therapy. Previously, the definition of response to immunosuppressive therapies in the setting of chronic sarcoidosis has relied on the measurement of improving or worsening organ function, such as measurements of left ventricular ejection fraction (LVEF) in patients with sarcoidosis and cardiac involvement, but this requires longer observation time and does not necessarily correlate with active inflammation. The current understanding of sarcoidosis pathology suggests that changes in organ function are dependent on ongoing granulomatous inflammation. Therefore, it would be intuitive to assess the treatment response by comparing FDG uptakes before and during therapy.

The European Respiratory Society (ERS) and the World Association for Sarcoidosis and Other Granulomatous Disorders (WASOG) recommend corticosteroid monotherapy as a first-line treatment in CS (11). In addition to long-term side effects, corticosteroid monotherapy is associated with high relapse rates after therapy cessation (12, 13). Additionally, in non-biologic and biologic disease-modifying anti-rheumatic drug (nb/bDMARD) therapies, relapse is reported in up to 88% of patients in whom cessation of therapy occurs after more than 2 years of treatment (14). These data would argue for longer-lasting DMARD therapies, but larger studies are needed to define the optimal regimen, combination therapies, and duration of therapy. Previous studies and reports have indicated that methotrexate, azathioprine, mycophenolate mofetil, and tumor necrosis factor inhibitor (TNFi) might be useful in cardiac sarcoidosis (15). TNFi, in particular, has received increased interest since it is normally well tolerated in the absence of unfavorable long-term side effects. Despite initial concerns about the association between high-dose infliximab and worsened heart failure, described in the setting of cardiomyopathy (16), recent studies in TNFi-treated patients with sarcoidosis-associated cardiac failure did not reveal a decrease in cardiac function (14, 17).

In this retrospective single-center study on 50 patients with CS, we evaluate the treatment efficacy of immunosuppressive regimens by sequential ^18^F-FDG PET/CT scans.

## Methods

### Study population

Patients were recruited from the Sarcoidosis Center of Excellence at the University Hospital Zurich between January 2016 and December 2020. Clinical or histological CS diagnosis was ascertained using the 2017 Japan Circulation Society expert consensus diagnostic criteria. Patients not fulfilling the diagnostic criteria but having a histological diagnosis of extracardiac sarcoidosis and characteristic cardiac ^18^F-FDG PET/CT findings were classified as having probable CS and were included in the study on the basis that they may represent an early stage of CS (in the absence of detectable structural myocardial damage). The study was approved by the Ethics Committee of the canton of Zurich (KEK-ZH 2014-0432).

### Clinical data collection

Baseline clinical and demographic characteristics at the time of diagnosis of CS (**Table 1**) were collected from the electronic medical records. Demographics investigated included age, sex, and weight. Medical history included histological evidence of non-necrotizing granuloma, extracardiac manifestations, and cardiac history at the time of diagnosis. Cardiac history included high-grade atrioventricular block (defined as AV block type Mobitz II or higher), cardiac syncope, sudden cardiac arrest, LVEF < 50%, signs of fibrosis on cardiac MRI, sustained ventricular arrhythmias, and implanted cardioverter defibrillator therapy. LVEF was considered at the time of diagnosis and final evaluation.

**Table 1:** Baseline characteristics of 50 cardiac sarcoidosis patients.

Demographics	Fulfill Japanese CS criteria (n = 34)	Total (n = 50)
Age, years	51.8 ± 11.9	51.3 ± 11.4
Women	18 (53%)	24 (48%)
Weight, kg	78.7 ± 19.6	76.7 ± 17.9
Pathology		
Positive extracardiac biopsy	26 (76%)	40 (80%)
Positive cardiac biopsy	4 (12%)	4 (8%)
Most common non-cardiac manifestation		
Lung	21 (62%)	32 (64%)
Lymph nodes	30 (88%)	45 (90%)
Cardiac history		
High-grade AV block	18 (53%)	19 (38%)
Syncope	9 (26%)	9 (18%)
Sudden cardiac arrest	3 (9%)	3 (6%)
LVEF < 50%	20 (59%)	20 (40%)
Sustained VT or VF	12 (35%)	12 (24%)
Cardiac fibrosis on MRI	26 (90%; n = 29)	27 (64%; n = 42)
ICD	32 (94%)	33 (66%)
LVEF	44.4% ± 13.7%	48.8% ± 13.3%
Comorbidities		
Lymphocytes < 1 * 10^9^/L	5 (15%)	11 (22%)
Diabetes	6 (17%)	6 (12%)
eGFR < 60 ml/min	1 (3%)	1 (12%)

Continuous variables are displayed as mean ± SD.

CS, cardiac sarcoidosis; VT, ventricular tachycardia; VF, ventricular fibrillation; ICD, implantable cardioverter-defibrillator; LVEF, left ventricular ejection fraction; MRI, magnetic resonance imaging; eGFR, estimated glomerular filtration rate.

### ^18^F-FDG-PET

Serial ^18^F-FDG PET/CT scans were performed before and approximately 6 months after therapy initiation or change of therapy and approximately every 6–12 months on stable therapy. PET/CT was performed in a 5-ring GE Discovery MI scanner, and data were analyzed using the GE Advanced Workstation 4.7 (GE Healthcare, Chicago, IL, USA).

To minimize the physiologic glucose uptake in myocardial cells, patients followed a strict carbohydrate-free diet to lower their blood glucose and insulin levels prior to the ^18^F-FDG PET/CT scan. Patients were asked to adhere to a carbohydrate-free, high-fat diet for 24 hours prior to the scan. On the day of the scan, blood sugar levels were measured, and heparin (50 IE/kg bodyweight) was injected 15 min prior to the injection of ^18^F-FDG adapted to body weight (approximately 200 MBq) to suppress the physiologic myocardial glucose metabolism (18). The examination was started approximately 60 min after the administration of ^18^F-FDG. A gated acquisition over the heart and a non-gated acquisition from neck to thighs was performed.

According to the Japanese Society of Nuclear Cardiology, the uptake was evaluated as pathological only if it showed a focal or focal on diffuse pattern (10). The left ventricular myocardium was evaluated in a standardized manner. First, the mean standardized uptake value (SUVmean) of the mediastinal blood pool was measured in the ascending aorta and multiplied by 1.5, defining the lower SUV threshold for the affected myocardium. The left myocardium was then marked, and the lower threshold value was applied in order to delineate only the affected myocardium, allowing for the calculation of the volume of the affected myocardium and SUVmean of the latter. Cardiac metabolic activity (CMA) was subsequently calculated by multiplying the affected myocardial volume of the left ventricular myocardium by the SUVmean (19, 20). The calculation is demonstrated in **Supplementary Figure 1**. For a number of ^18^F-FDG PET/CT examinations (n = 7), it was not possible to retrospectively calculate CMA values due to missing data, and for these, only originally reported SUVmax values were included. The initial PET/CT evaluation was undertaken by a specialist in nuclear medicine and cardiology. Subsequent re-evaluation and assessment of CMA were performed by a dual-certified specialist in radiology and nuclear medicine.

### Statistical analysis and outcome measures

**Figure 1:**
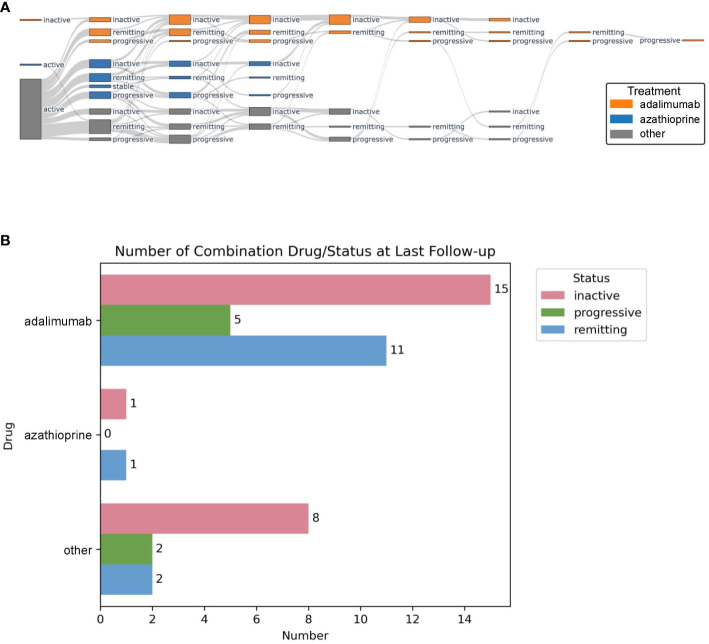
Sankey plot showing disease state and treatment group at each follow-up PET/CT scan. Adalimumab-treated patients are depicted in orange, azathioprine in blue, and others in gray **(A)**. Disease status at last follow-up PET/CT scan for adalimumab, azathioprine, and other treatments **(B)**.

Response to different immunosuppressive therapies was retrospectively assessed by cardiac ^18^F-FDG uptake. Left ventricular myocardial SUVmax and CMA values were used to characterize treatment response. Active disease was defined as an SUVmax > 3.5. On follow-up PET/CT scans, patients with active disease were classified into stable (SUVmax ± 0.2), progressive if the value was increased by >0.2 compared to the previous, and remitting if the value was decreased by >0.2 compared to the previous but still above 3.5. Each observation (including baseline and subsequent follow-up scans for each patient) was included in a group defined by the patient’s treatment at the time of the first follow-up and subsequently analyzed according to status/response at the time of the last follow-up. Treatment groups were defined by therapy at the time of first follow-up, with i) azathioprine, ii) adalimumab, or iii) other (when neither of the two drugs was administered). Status/response groups are defined as described above. Patients without follow-up were not included. For the Sankey plot (**Figure 1A**), at each follow-up, patients with the same treatment and status were grouped together, and consecutive follow-ups from the same patient were linked. Plotly’s python library was used to plot the Sankey graph. The last follow-up for each patient was selected to explore the final distribution between status and treatment using Matplotlib (**Figure 1B**).

In addition, LVEF before and at the last follow-up under TNFi was compared. Continuous variables were reported as mean and standard deviation. Pre- and post-treatment differences were analyzed by a paired samples t-test. One-way analysis of variance (ANOVA) followed by Tukey’s multiple comparisons as a *post hoc* test for multiple comparisons was used to compare mean SUVmax, CMA, and prednisone dosage in the different treatment groups. Categorical variables are presented as frequencies and percentages. A p-value of <0.05 was considered statistically significant. Statistical analyses and data presentations were performed using Prism (GraphPad Software Inc.).

## Results

### Baseline characteristics

Between January 2016 and December 2020, 50 patients with a diagnosis of CS, or probable CS, were referred to our interdisciplinary ambulatory Sarcoidosis Center of Excellence, of which 34 (68%) fulfilled the 2017 Japan Circulation Society expert consensus CS diagnostic criteria (10). The remaining 16 (32%) patients had histologically confirmed extracardiac sarcoidosis and showed a typical pattern of myocardial ^18^F-FDG uptake but did not fulfill the above-mentioned criteria (**Table 1**). From 45 of the 50 patients (90%), baseline and at least one follow-up PET/CT scan were available. The mean interval between the first and last follow-up PET/CT scans from diagnosis was 6.7 ± 3 and 20.5 ± 12.8 months, respectively.

The mean age of the patients was 51.3 ± 11.4 years. The most common non-cardiac sarcoidosis manifestations were in the lung and lymph nodes (**Table 1**). Four patients fulfilling the CS diagnostic criteria had isolated cardiac sarcoidosis. The majority of patients presented with cardiac symptoms (n = 34; 68%), commonly with high-grade AV block (n = 19; 38%). Two-thirds of the patients received a prophylactic implantable cardioverter-defibrillator (ICD) therapy. The cardiac history and treatment-relevant comorbidities are summarized in **Table 1**. The 16 sarcoidosis patients not fulfilling the CS criteria did not have any cardiac history and were diagnosed on screening myocardial PET/CT scan for cardiac involvement.

### Immunosuppressive management

From the 45 patients with baseline and follow-up PET/CT scans available after initial diagnosis, 29 (64%) patients received high-dose corticosteroids (methylprednisolone pulse with 500–1,000 mg for 3 days followed by prednisone 40–80 mg daily for at least 4 weeks), 12 (27%) patients were treated with low-dose corticosteroids (20–30 mg daily for at least 4 weeks), and four patients (9%) remained steroid-free. In addition, 37 (82%) patients received “steroid-sparing” immunosuppressive therapy concomitantly. According to current guidelines (11), nbDMARDs were used as first-line therapy unless patients had contraindications. As such, patients were treated as follows: nbDMARDs in 19 (42%) with azathioprine (mean 132 ± 32 mg daily), three (7%) with roflumilast (mean 500 ± 0 μg daily), two (4%) with methotrexate (mean 13.75 ± 1.25 mg subcutaneous (s.c.) weekly), one (2%) with mycophenolate mofetil (MMF; 2 g daily), and one (2%) with tofacitinib (10 mg daily); bDMARDs in 10 (22%) with adalimumab (nine patients 40 mg weekly s.c. and one patient 40 mg every other week) and one (2%) with infliximab (5 mg per kilogram body weight every 8 weeks intravenously); no nb/bDMARDs were administered in eight patients (18%). The decision for initial corticosteroid and DMARD therapy was open to physician choice and based on comorbidities. Furthermore, the COVID pandemic influenced the initial steroid therapy by avoidance of in-hospital methylprednisolone pulse therapy. Corticosteroids were tapered every 4 weeks aimed at reaching a dose ≤ 10 mg after 4–6 months of therapy. At the time of the first follow-up PET/CT scan, corticosteroids had been tapered to 8.1 ± 6.8 mg.

During follow-up, nb/bDMARDs were changed based on treatment response and treatment-related adverse effects. The average time on each medication was 11.4 ± 6.6 months. **Figure 1A** graphically shows disease status for the largest treatment groups with adalimumab (n = 10, 22%) *vs.* azathioprine (n = 19, 42%) compared to all other treatments at each follow-up PET/CT. During follow-up, the following therapeutic switches were undertaken: 12 from azathioprine to adalimumab (seven because of insufficient therapy response and five because of persistent severe lymphopenia), nine from treatments other than azathioprine to adalimumab (all because of insufficient therapy response), and six from azathioprine to treatments other than adalimumab (once again because of insufficient therapy response), and one patient stopped taking adalimumab because of side effects, later re-initiated during follow-up because of relapsing disease. At the first follow-up PET/CT scan, 12 patients (64%) receiving azathioprine, eight patients (80%) receiving adalimumab, and six patients (76%) without DMARD reached inactive or remitting disease (**Table 2**). All 15 patients receiving adalimumab as second-line treatment, and 83% of the patients receiving nbDMARDs (three methotrexate, two MMF, and one azathioprine) reached inactive or remitting disease. In contrast, all three patients who, based on patient preference, stopped their first-line therapy had progressive disease at the next follow-up. At the last follow-up, 31 patients (69%) were on adalimumab. Of these, 84% showed an inactive or remitting disease state (**Figure 1B**).

**Table 2:** Response to first- and second-line therapy.

	Inactive	Remitting	Stable	Progressive	ΔSUV	PDN (mg)
First-line						
Azathioprine (n = 19)	6	6	1	6	−2.4 ± 5.6	9.9 ± 5.2
Adalimumab (n = 10)	3	5	0	2	−5.1 ± 9.1	7.3 ± 5.1
No DMARD (n = 8)	1	5	0	2	−3.5 ± 5.9	10 ± 10
Second-line						
nbDMARD (n = 6)	3	2	0	1	−1.7 ± 6.9	9.1 ± 6.1
Adalimumab (n = 15)	10	5	0	0	−4.2 ± 4.2	5.3 ± 1.2
No DMARD (n = 3)	0	0	0	3	+4.5 ± 3.1	0 ± 0

PDN, prednisone; DMARD, disease-modifying anti-rheumatic drug; nbDMARD, non-biologic disease-modifying anti-rheumatic drug.

Serious adverse events occurred in five patients. One patient on methotrexate died because of cardiac arrest and another on adalimumab because of a cerebrovascular insult. The remaining three patients experienced ventricular tachycardia (two on roflumilast and one on adalimumab). All patients with cardiac events had signs of cardiac fibrosis on MRI. No other serious adverse events were observed.

### Suppression of myocardial ^18^F-FDG uptake at first follow-up

**Figure 2:**
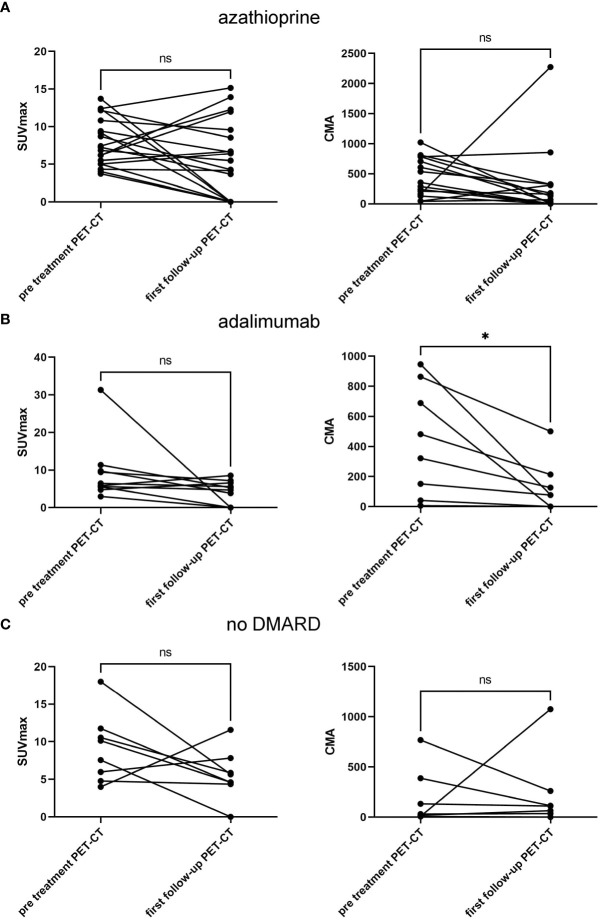
Treatment response of CS treated with azathioprine **(A)**, with adalimumab **(B)**, and without nb/bDMARD **(C)** showing SUVmax (left column) and CMA values (right column) pretreatment and at first follow-up PET/CT. Paired t-test was used to assess p-values; *p < 0.05, ns, not significant. CS, cardiac sarcoidosis; nb/DMARD, non-biological and biological disease-modifying anti-rheumatic drug; CMA, cardiac metabolic activity.

At the first follow-up PET/CT scan, there was a reduction in SUVmax from 7.9 ± 3.1 to 5.7 ± 5 and in CMA from 449 ± 308 to 311 ± 568 in the azathioprine-treated group (**Figure 2A**). However, none of these reductions reached statistical significance (p = 0.11 and p = 0.46). The adalimumab-treated group showed a statistically significant reduction in CMA from 437 ± 344 to 125 ± 158 (p = 0.026) but not for SUVmax, from 9.3 ± 7.7 to 4.2 ± 3 (p = 0.13) (**Figure 2B**). The subgroup without nb/bDMARDs showed a non-significant reduction in SUVmax from 9.1 ± 4.3 to 5.6 ± 3.1 (p = 0.15) and a slight increase in CMA from 193 ± 263 to 237 ± 351 (**Figure 2C**). We did not detect any difference between high-dose and low-dose corticosteroid-treated patients (data not shown). At the timepoint of the first follow-up PET/CT scan, the prednisone dose was lowest in the adalimumab-treated group 7.3 ± 5.1 mg compared to azathioprine 9.9 ± 5.2 mg and 10 ± 10 mg in the subgroup without nb/bDMARDs. However, these differences did not reach statistical significance. The other treatment groups were too small for meaningful statistical sub-analyses.

### Myocardial ^18^F-FDG uptake on follow-up PET/CT scans according to immunosuppressive management

**Figure 3:**
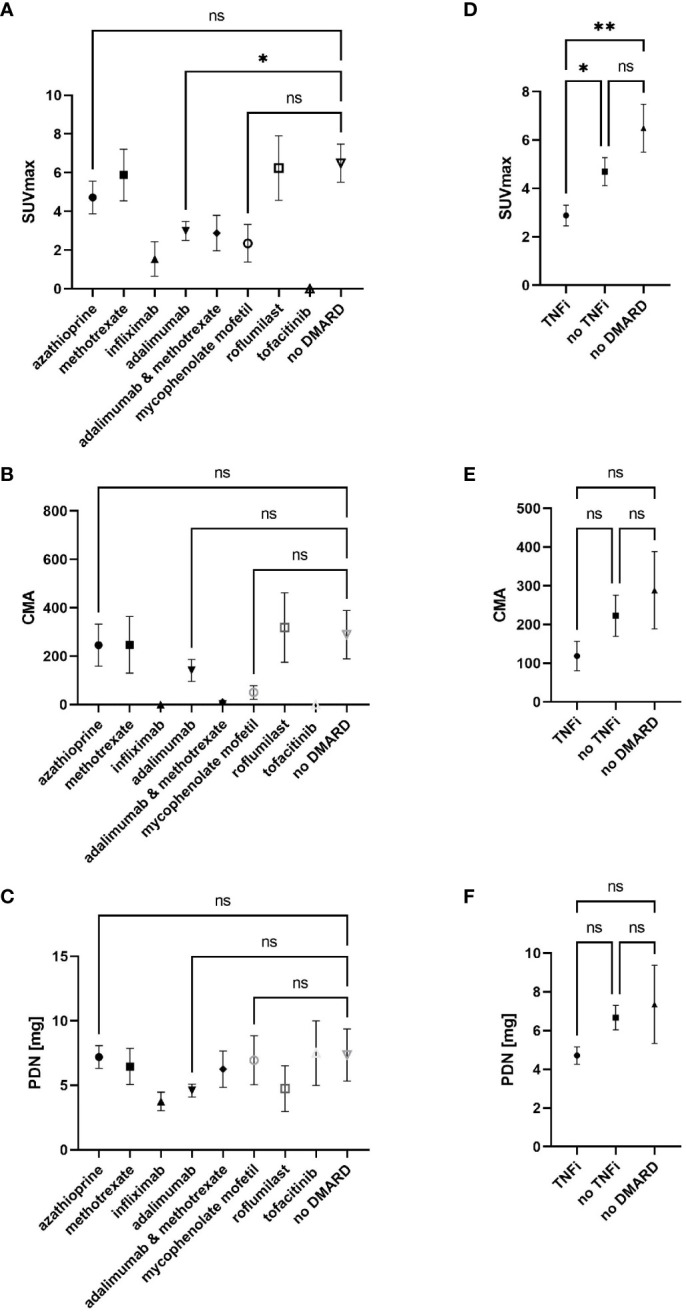
SUVmax **(A)** and CMA **(B)** values as well as prednisone dose **(C)** at all 143 follow-up PET/CT scans according to nb/bDMARD used at timepoint of follow-up PET/CT as well as comparison of TNFi containing and not containing DMARD therapies with no DMARD for SUVmax **(D)**, CMA **(E)**, and prednisone dose **(F)**. One-way analysis of variance (ANOVA) followed by Tukey’s multiple comparisons as *post hoc* test for multiple comparisons was used to assess p-values; *p < 0.05, **p < 0.01, ns, not significant. CMA, cardiac metabolic activity; nb/DMARD, non-biological and biological disease-modifying anti-rheumatic drug; TNFi, tumor necrosis factor inhibitor.

In total, from 45 patients with at least one follow-up, 143 PET/CT scans were analyzed. **Table 3** shows the number of follow-up PET/CT scans as well as the therapeutic regimen and mean prednisone dosage at the timepoint of each follow-up PET/CT scan. The mean time between the two follow-ups was 6.6 ± 2.4 months. The most frequent therapies used were adalimumab monotherapy (50 follow-up PET/CT in 30 patients) and azathioprine (33 follow-up PET/CT in 21 patients). Comparing SUVmax values at all follow-up PET/CT scans between the different therapies, a statistically significant lower SUVmax was observed for the adalimumab-treated group compared to the group without nb/bDMARDs (**Figure 3A**). A similar trend to a lower activity could be observed with infliximab, mycophenolate mofetil, tofacitinib, or a combination of adalimumab and methotrexate. Activity assessed by CMA showed comparable results but without statistical significance (**Figure 3B**). Prednisone dose was comparable in all treatment groups with a trend to lower doses for infliximab and adalimumab (**Figure 3C**). Comparing all TNFi regimens with nbDMARD treatments and prednisone-only-treated patients, we observed a statistically significant lower SUVmax for TNFi (**Figure 3D**). A similar trend was visible for CMA without reaching statistical significance (**Figure 3E**). Prednisone doses were comparable in all three groups with a trend to lower doses in patients under TNFi therapy (**Figure 3F**). Almost all patients responded to second-line DMARD therapies (**Table 2**).

**Table 3:** Patient follow-up.

	Total	AZA	MTX	INF	ADA	ADA/MTX	MMF	ROF	TOF	No	PDN (mg)
Follow-up PET 1	45	19	2	1	10	0	1	3	1	8	8.1 ± 6.8
Follow-up PET 2	37	9	1	2	11	2	3	3	1	5	5.6 ± 4.2
Follow-up PET 3	26	5	3	1	11	0	3	2	0	1	5.1 ± 3.8
Follow-up PET 4	17	0	3	0	7	2	2	2	0	1	4.3 ± 2.9
Follow-up PET 5	8	0	1	0	5	1	0	0	0	1	3.75 ± 3.1
Follow-up PET 6	7	0	2	0	3	1	0	0	0	1	5 ± 1.3
Follow-up PET 7	2	0	0	0	2	0	0	0	0	0	5 ± 0
Follow-up PET 8	1	0	0	0	1	0	0	0	0	0	5 ± 0
**Total**	**143**	**33**	**12**	**4**	**50**	**6**	**9**	**10**	**2**	**17**	**5.9 ± 5**

AZA, azathioprine; MTX, methotrexate; INF, infliximab; ADA, adalimumab; MMF, mycophenolate mofetil; ROF, roflumilast; TOF, tofacitinib; No, no corticosteroid-sparing therapy; PDN, prednisone.

### Change in LVEF under TNFi therapy

**Figure 4:**
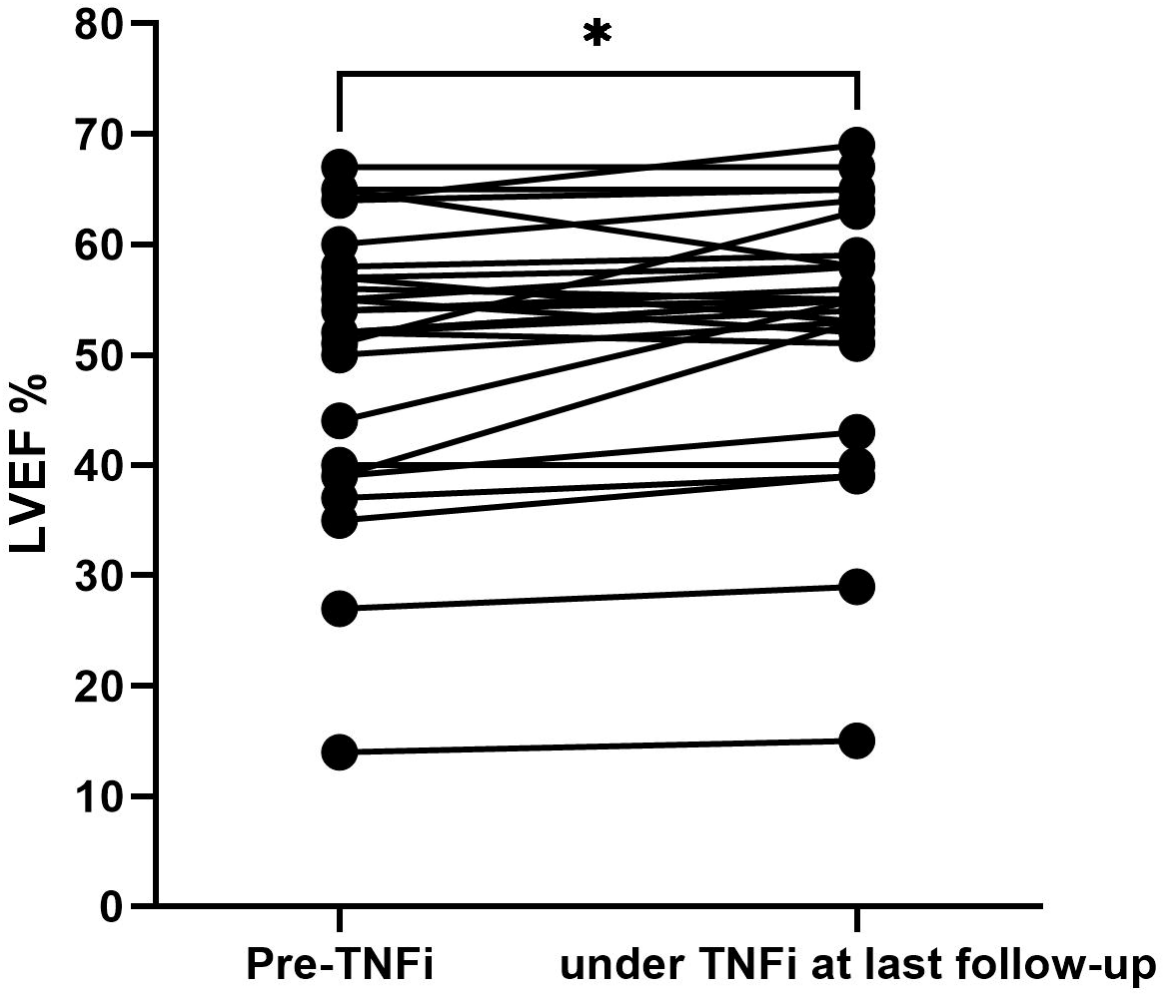
Pre- and last follow-up under TNFi LVEF measurements. Paired t-test was used to assess p-values; *p < 0.05. TNFi, tumor necrosis factor inhibitor; LVEF, left ventricular ejection fraction.

There is concern that TNFi may potentially worsen heart failure in patients with reduced LVEF. For that reason, we compared the last LVEF before TNFi with the last follow-up LVEF on treatment, both assessed by transthoracic echocardiogram. Data were available from 28 patients (85%) before and during TNFi treatment. The mean duration of TNFi was 13.6 ± 9.2 months. Overall, LVEF was stable or even improved in patients receiving TNFi (pre TNFi 51% versus 53% at the last follow-up on TNFi, p = 0.012) (**Figure 4**). Of note, six patients had an LVEF < 40% and two patients had an LVEF < 30% at the time of TNFi initiation. All of them showed stable or improved LVEF at the time of the last follow-up PET/CT scan.

## Discussion

In this retrospective single-center study, we present outcomes of immunosuppressive regimens as assessed by myocardial ^18^F-FDG uptake in a cohort of CS patients followed up for 20.5 ± 12.8 months. We observed a reduction of SUVmax and CMA with most of the nb/bDMARDs used as first- and second-line agents. However, only adalimumab-based therapy was associated with a statistically significant reduction in cardiac metabolic activity at the first follow-up PET/CT scan. Twenty-one patients (47%) were switched to adalimumab-based therapies during follow-up, and 84% of the adalimumab-treated patients reached inactive (n = 15, 48%) or remitting (n = 11, 35%) disease states. Considering all follow-up PET/CT scans analyzed, TNFi also showed significantly better suppression of myocardial ^18^F-FDG uptake compared to other treatment regimens. Furthermore, we observed that TNFi treatment was safe even in patients with severely reduced LVEF. A slight but statistically significant improvement in LVEF was observed under TNFi. This finding is in line with previous reports that have also shown favorable response rates and safety data for TNFi in CS (14, 17, 21, 22). Given the high relapse rates of CS after corticosteroid tapering and the side effects associated with long-term corticosteroid therapy, our data support a potential strategy of initiating immunosuppression with TNFi in addition to corticosteroids immediately after diagnosis of CS. TNF-alpha is an important mediator of macrophage activation and granulomatous inflammation, and TNFi has been shown to be an effective treatment for patients with systemic and CS (14, 17, 21–24). Given the better response rate as evaluated by PET/CT as well as the safety shown in our study, it could be considered as a first-line therapy, especially in patients with comorbidities and without contraindications for TNFi therapy.

Based on the results of the Anti-TNF Therapy Against Congestive Heart Failure (ATTACH) trial showing an association between high-dose infliximab and worsened heart failure, TNFi is contraindicated in New York Heart Association class III and IV heart failure (16). In patients with cardiac sarcoidosis and reduced LVEF, TNFi should be used with caution and under close observation of LVEF and clinical signs of heart failure. In our cohort, none of the patients with reduced LVEF showed a worsening of heart failure, which suggests that TNFi therapy could be used also in the subgroup of CS patients with reduced LVEF. This finding, however, must be considered in light of the limited follow-up time of this study.

In addition to SUVmax, we used CMA as a parameter to assess therapeutic response. High CMA has previously been shown to be a significant risk marker for cardiac events (20). Assessing the therapeutic response with these two parameters might help guide treatment decisions before cardiac events become apparent. It is however not known if a complete resolution of myocardial ^18^F-FDG uptake is mandatory to avoid the development of myocardial fibrosis and subsequent cardiac events or if a low myocardial ^18^F-FDG uptake could be tolerated. Long-term comparative studies are necessary to answer these questions.

Our study has several inherent limitations related to the retrospective, observational design as well as to the heterogeneity of initial therapy. The response to the different treatment regimens used in our cohort cannot be directly compared due to the large heterogeneity of initial treatments and switching of therapy in case of side effects or non-response. It is however very representative of real-world clinical decision making and therefore relevant to current clinical practice.

In conclusion, our study corroborates the efficacy of TNFi in suppressing cardiac inflammation and positively influencing cardiac function. In case of unresponsiveness or side effects to nbDMARDs, TNFi is an effective alternative. In some patients with comorbidities or contraindications to alternative therapies, it could be considered as a first-line “steroid-sparing” agent. Larger prospective and randomized trials will be necessary to systematically compare the efficacy of TNFi against other immunosuppressive therapies as well as the efficacy of regular treatment monitoring with ^18^F-FDG PET/CT.

## Data availability statement

The raw data supporting the conclusions of this article will be made available by the authors, without undue reservation.

## Ethics statement

The studies involving humans were approved by Ethical Committee Zurich (KEK-ZH 2014-0432). The studies were conducted in accordance with the local legislation and institutional requirements. The participants provided their written informed consent to participate in this study.

## Author contributions

LF: Conceptualization, Data curation, Formal Analysis, Investigation, Methodology, Project administration, Validation, Writing – original draft, Writing – review & editing. JaS: Data curation, Formal Analysis, Investigation, Methodology, Visualization, Writing – original draft, Writing – review & editing. ES: Data curation, Visualization, Writing – review & editing. AY: Data curation, Writing – review & editing. DA: Data curation, Writing – review & editing. JuS: Data curation, Writing – review & editing. CG: Data curation, Writing – review & editing. RB: Data curation, Writing – review & editing. DF: Data curation, Writing – review & editing. AK: Conceptualization, Data curation, Investigation, Methodology, Supervision, Visualization, Writing – original draft, Writing – review & editing. JN: Conceptualization, Data curation, Investigation, Methodology, Supervision, Writing – original draft, Writing – review & editing.

## Supplementary material

Supplementary Figure 1 Calculation of cardiac metabolic activity (CMA) quantification calculated according to the depicted flow-chart. Shown is a representative sample.
