# Supplementary figures and images for: Assessment of treatment response in cardiac sarcoidosis based on myocardial 18F-FDG uptake
Source: Front Immunol. 2023 Nov 24;14:1286684. doi: 10.3389/fimmu.2023.1286684 (PMC10704456; doi:10.3389/fimmu.2023.1286684)

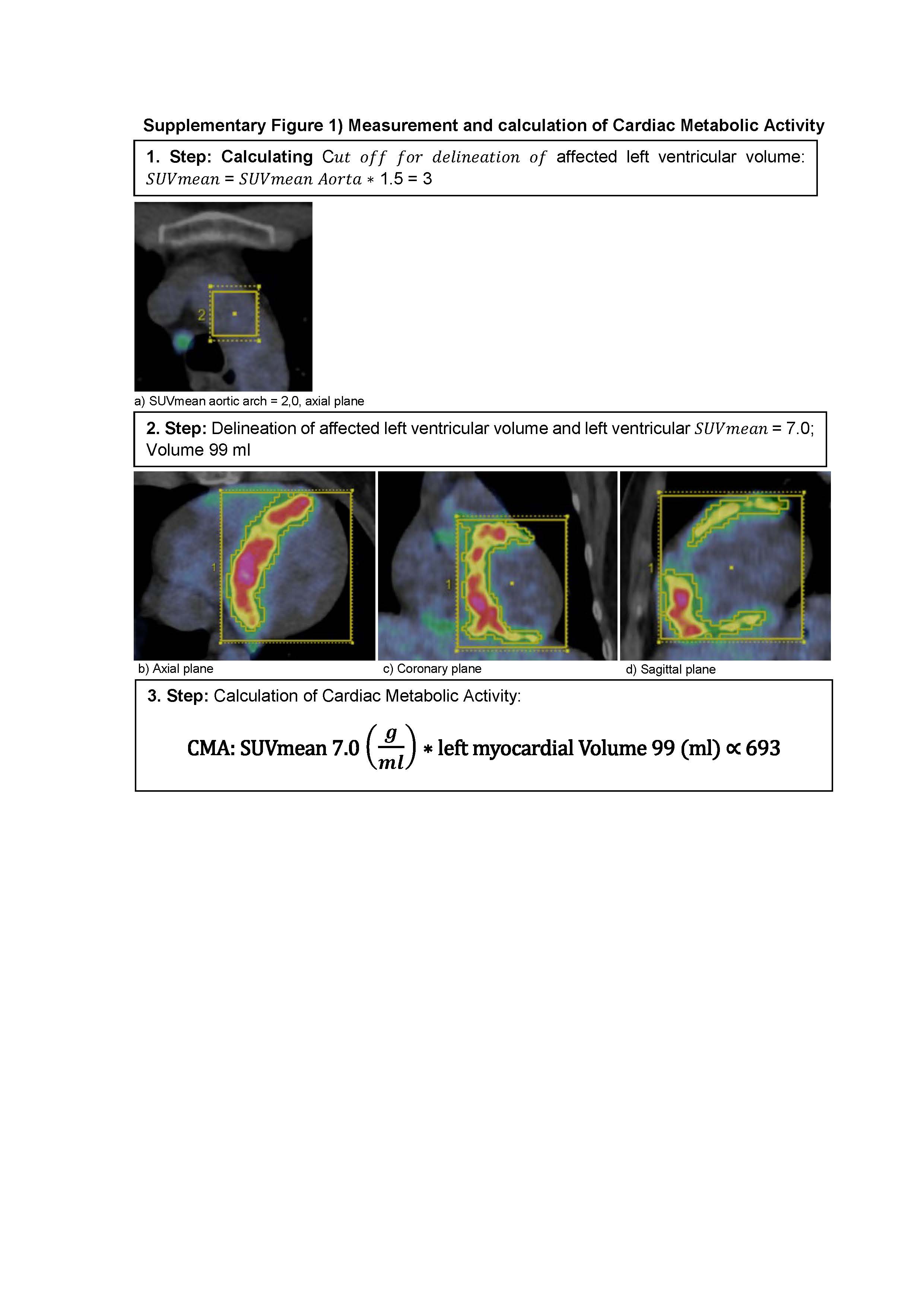

Supplement: Supplementary Figure 1 — Calculation of cardiac metabolic activity (CMA) quantification calculated according to the depicted flow-chart. Shown is a representative sample. [file Image_1.jpg]
